# Examining buprenorphine diversion through a harm reduction lens: an agent-based modeling study

**DOI:** 10.1186/s12954-023-00888-6

**Published:** 2023-10-17

**Authors:** Joëlla W. Adams, Michael Duprey, Sazid Khan, Jessica Cance, Donald P. Rice, Georgiy Bobashev

**Affiliations:** 1 RTI International, Research Triangle, NC USA; 2 Division of Infectious Disease, Department of Medicine, Alpert Medical School of Brown University, Providence, RI USA

**Keywords:** Opioids, Modeling, Overdose, Harm reduction

## Abstract

**Background:**

Recent policies have lessened restrictions around prescribing buprenorphine-naloxone (buprenorphine) for the treatment of opioid use disorder (OUD). The primary concern expressed by critics of these policies is the potential for buprenorphine diversion. However, the population-level effects of increased buprenorphine diversion are unclear. If replacing the use of heroin or fentanyl, use of diverted buprenorphine could be protective.

**Methods:**

Our study aim was to estimate the impact of buprenorphine diversion on opioid overdose using an agent-based model calibrated to North Carolina. We simulated the progression of opioid misuse and opioid-related outcomes over a 5-year period. Our *status quo* scenario assumed that 50% of those prescribed buprenorphine diverted at least one dose per week to other individuals with OUD and 10% of individuals with OUD used diverted buprenorphine at least once per week. A *controlled prescription only* scenario assumed that no buprenorphine would be diverted, while an *increased diversion* scenario assumed that 95% of those prescribed buprenorphine diverted and 50% of individuals with OUD used diverted buprenorphine. We assumed that use of diverted buprenorphine replaced the use of other opioids for that day. Sensitivity analyses increased the risk of overdose when using diverted buprenorphine, increased the frequency of diverted buprenorphine use, and simulated use of diverted buprenorphine by opioid-naïve individuals. Scenarios were compared on opioid overdose-related outcomes over the 5-year period.

**Results:**

Our *status quo* scenario predicted 10,658 (credible interval [CI]: 9699–11,679) fatal opioid overdoses. A scenario simulating *controlled prescription only* of buprenorphine (i.e., no diversion) resulted in 10,741 (9895–11,650) fatal opioid overdoses versus 10,301 (9439–11,244) within a scenario simulating *increased diversion*. Compared to the *status* quo, the *controlled prescription only* scenario resulted in a similar number of fatal overdoses, while the scenario with *increased diversion* of buprenorphine resulted in 357 (3.35%) fewer fatal overdoses. Even when increasing overdose risk while using diverted buprenorphine and incorporating use by opioid naïve individuals, increased diversion did not increase overdoses compared to a scenario with no buprenorphine diversion.

**Conclusions:**

A similar number of opioid overdoses occurred under modeling conditions with increased rates of buprenorphine diversion among persons with OUD, with non-statistical trends toward lower opioid overdoses. These results support existing calls for low- to no-barrier access to buprenorphine for persons with OUD.

**Supplementary Information:**

The online version contains supplementary material available at 10.1186/s12954-023-00888-6.

## Background

The current strategy to address the opioid epidemic within the USA places harm reduction and increased access to the three FDA-approved medications for opioid use disorder (MOUD; methadone, buprenorphine, and naltrexone) in the center of the nation’s efforts. During the COVID-19 pandemic, increased flexibility around MOUD prescribing such as telehealth visits to initiate treatment and take-home methadone were introduced to facilitate access to care [1]. In January 2023, the government removed the federal requirement for practitioners to have a Drug Addiction Treatment Act waiver (referred to as the “X-waiver”) to prescribe buprenorphine for the treatment of opioid use disorder and changed limitations on the number of patients prescribed buprenorphine per provider. This will increase the number of practitioners able to prescribe buprenorphine and is intended to help address barriers to accessing OUD treatment. Buprenorphine treatment is shown to halve the risk of opioid overdose for those in formal treatment and is protective even when used outside of formal treatment settings [2–4]. Buprenorphine, compared to placebo, improves retention in treatment and, at high doses, has similar retention and abstinence rates to methadone treatment [5]. As a partial opioid agonist, buprenorphine has a superior safety profile compared to full *mu* opioid receptor agonists such as oxycodone with respect to respiratory depression and fatal overdose [6]. Co-formulation with naloxone decreases the likelihood of injecting, the riskiest method of consumption, and co-formulated buprenorphine is standard treatment for OUD.

Concerns have been raised that increased flexibility in MOUD prescribing and the removal of the waiver will increase the diversion of buprenorphine which, in turn, could increase overdose deaths [6, 7]. Overdose might be more likely for individuals using MOUD without physician oversight, as the probability of concurrent use of other substances such as benzodiazepines and alcohol is increased, doses are self-administered, and medical monitoring is not possible [8]. In addition, diverted buprenorphine can be misused for euphoria and poses a risk to pediatric populations if accidentally ingested [9, 10]. However, studies suggest that most diverted buprenorphine in the USA is used by people who already have an opioid dependence to reduce their health risks from opioid use, stave off withdrawal, or bridge gaps in treatment, rather than for euphoria [11–13]. Furthermore, self-treatment with diverted buprenorphine will lower overdose risk if individuals use diverted buprenorphine instead of heroin and fentanyl [2]. Therefore, it is unclear whether increased buprenorphine diversion will lead to increases in overdose deaths.

Questions remain regarding the population-level effects of diverted buprenorphine on non-fatal and fatal opioid overdose. Unfortunately, long-term impacts of policies increasing the likelihood of buprenorphine diversion will not be known for years. In addition, it is not feasible or ethical to vary the rate of buprenorphine diversion using a traditional study design such as a randomized controlled trial. An agent-based model can simulate individual-level interactions (i.e., sharing of buprenorphine) while providing causal estimates at the population level (i.e., overdose incidence) to provide policy insights quickly. Within this analysis, we used an agent-based model as a timely policy tool to provide insight on the potential impact of changing regulations which could increase buprenorphine diversion.

## Methods

### Analytic overview

Using an agent-based model calibrated to reproduce opioid overdose and mortality rates in North Carolina from 2010 to 2019, we simulated the use and misuse of prescription opioids (PO), and use of heroin/fentanyl, the progression of OUD, treatment with MOUD, and peer networks to compare how differing levels of buprenorphine diversion impacted opioid-related outcomes. Model scenarios were run with a 5-year timeframe under conditions reflective of North Carolina with an assumption that recent historical trends would continue (i.e., high levels of illicitly manufactured fentanyl contaminating the drug supply, increasing trend in overdose deaths).

A 5-year time frame was used to provide insight into the potential long-term effects of increased buprenorphine diversion and to account for individuals who transition in and out of treatment for OUD and opioid use or misuse. We compared three model scenarios: (1) a *status quo* scenario based on existing research and reflecting a moderate level of buprenorphine diversion (50% of those prescribed divert at least a portion of their doses, and 10% of individuals with OUD use diverted buprenorphine); (2) *controlled prescription only* (no diversion among those prescribed and only individuals prescribed buprenorphine use the medication); and (3) *increased diversion* (90% of those prescribed buprenorphine divert 1 to 2 doses per week, and 50% of individuals with OUD use diverted buprenorphine). Metrics compared across scenarios included the number of non-fatal and fatal opioid overdoses as well as the number of individuals who ever used diverted buprenorphine.

### Model overview

The Comprehensive Opioid Policy Agent-Based Model (ABM) simulates multiple pathways of opioid use, including starting with the use of PO for chronic or acute pain, and transitions from other substances into opioids. Misused opioids include POs, heroin/fentanyl, and MOUD including buprenorphine. Agent interactions include dealer–user relationships; accessing POs through emergency departments, pharmacies, and other healthcare settings; and user/peer networks. This model is intended for use in examining long-term effects of prevention and treatment interventions on opioid misuse. Table 1 summarizes selected key parameters and sources. The accompanying Additional file 1 provides detailed information on modeling processes, parameter sources, and assumptions related to this analysis.

**Table 1:** Selected key model parameters

Parameter	Value (distribution)	Source
*Pain and Opioid Use States*		
Probability of an agent developing acute pain over 12 months	15%	Mikosz et al. [14]
Percent of agents presenting with an acute condition (postsurgical or physical trauma) who receive a prescription opioid	22%	Mikosz et al. [14]
The initial daily mean MME needed to treat acute pain (e.g., underlying pain level)	6 MME for 70%, 30 MME for 30%	Howard et al. [15]
The initial daily mean MME prescription picked up at pharmacy for acute pain	30 MME (normal, 10 SD)	Mikosz et al. [14]
Length of prescription in days for acute pain	7 days (3–7 days)	Mundkur et al. [16], Dowell et al. [17]
Probability that an individual physician refills a prescription opioid or increases dosage of an opioid to a patient reporting acute pain after initial prescription	25% (uniform, 12–30%)	Mundkur et al. [16]
Agents experiencing chronic pain (with or without opioid use). Estimate specific for NC	1,671,000	Zelaya et al. [18]
Percent of agents presenting with chronic pain who receive a prescription opioid	30%	Mathieson et al. [19]
The initial daily mean MME needed to treat chronic pain (e.g., underlying pain level)	52 MME (normal, 10 SD)	Naliboff et al. [20]
The initial daily mean MME prescription picked up at pharmacy for chronic pain	50 MME (normal, 10 SD)	Dowell et al. [17]
Length of prescription in days for chronic pain	28 days (7–28 days)	Dowell et al. [17]
Probability that an individual physician refills a prescription opioid or increases dosage of an opioid to a patient reporting chronic pain after initial prescription	90%	Dowell et al. [17], HHS [21], FDA [22]
*Physician and Prescription Behaviors*		
Percentage of prescriptions complying with cap on dosage (< 90 MME/day)	92.7%	Centers for Disease Control and Prevention. 2019 Annual Surveillance Report of Drug-Related Risks and Outcomes [23]
Percentage of physicians complying with prescription drug monitoring laws	77.5% (75–80%)	Borrelli et al. [24], Hung et al. [25]
Probability that an individual physician refills an existing opioid prescription or increases dosage of an opioid to a patient reporting continued acute or chronic pain	Acute pain: mean 25%, SD 10% Chronic pain: mean 90% SD 10%	Dowell et al. [17], Mundkur et al. [16]
Mean percent increase in prescribed dose if patient reports continued pain	25%	Gallagher et al. [26]
*Treatment for Opioid Use Disorder*		
Annual probability of seeking treatment for agents who misuse opioids or use heroin	25% (5–45%)	NSDUH 2019, Fox et al. [27], Blanco et al. [28]
Estimated number of individuals entering treatment for opioid use disorder per year	5,167	Knopf [29]
Number of people with an opioid use disorder that are currently in treatment	24,227	N-SSATS 2019
Probability of receiving methadone once somebody starts treatment	0.59	N-SSATS 2019
Probability of receiving buprenorphine once somebody starts treatment	0.38	N-SSATS 2019
Probability of receiving naltrexone once somebody starts treatment	0.03	N-SSATS 2019
Overdose rate for an agent actively taking methadone	2 per 100 person-years	Sordo et al. [3]
Overdose rate for an agent actively taking buprenorphine	2.08 per 100 person-years	Morgan et al. [30]
Overdose rate for an agent with a recent (less than one month old) injectable naltrexone	3.85 per 100 person-years	Morgan et al. [30]
Annual probability of methadone treatment cessation, agent goes back to most recent active use state	0.55 by 1 year	Timko et al. [31], Soyka et al. [32]
Annual probability of buprenorphine treatment cessation, agent goes back to most recent active use state	0.31 by one month, 0.735 by 1 year	Morgan et al. [33]
Annual probability of naltrexone treatment cessation, agent goes back to most recent active use state	0.52 by one month, 0.95 by 1 year	Morgan et al. [33]
*Overdose*		
Algorithm of probability of overdose by MME dose	Varies	Dunn et al. [34], Calibrated to NCDHHS
Multiplier of risk of overdose related to use of heroin compared to someone solely using prescription opioids (calibrated to reflect NC OD data)	1.7	Calibrated to NCDHHS data
Probability of fatal overdose, conditional on experiencing overdose	0.17	Calibrated to NCDHHS data
Multiplier of risk of fatal overdose related to use of heroin compared to someone solely using prescription opioids (calibrated to reflect NC OD data)	1.5	Calibrated to NCDHHS data
Probability of naloxone being available to any agent	27.6%	https://naloxonesaves.org/community-distribution-of-naloxone/, NCDHHS
Probability of reversal after use of naloxone during an overdose event	87.5% (75–100%)	Clark et al. [35]
*Buprenorphine Diversion*		
Probability of sharing/selling buprenorphine conditional on being prescribed	50%	Lofwall 2015 [6], Kenney 2017 [36]
Probability of using diverted buprenorphine conditional on using illicit opioids	10%	Genberg 2013 [37], Yokell 2011 [38], Fox 2015 [39], Novak 2015 [40]
Probability of using diverted buprenorphine vs. illicit opioid conditional on having access to diverted buprenorphine	15%	Carlson et al. [2]
Probability of sourcing diverted buprenorphine from a friend/peer vs. dealer	80%	Kenney 2017 [36], Larance et al. [41]
Probability of overdose while using diverted buprenorphine	2.08 per 100 person-years	Morgan et al. [30]
Assortative mixing parameter for agents who have been prescribed buprenorphine and/or have used diverted buprenorphine	45%	Experimental parameter

### Opioid use pathways

Agents can develop OUD through pathways related to the misuse of opioid medications prescribed for acute or chronic pain or through the initiation of opioid misuse for euphoria. Each agent has three internal state variables—*desire*, *tolerance*, and *satiation*—which change over time in response to the use of opioids. *Desire* reflects the morphine milligram equivalent (MME) sought out by the agent. While *desire* is related to the amount of MME needed to reach satiation, it can also incorporate the seeking of additional MME for recreational use or increased MME related to tolerance. *Tolerance* is assumed to be 0 for an opioid-naïve individual. Patients develop *tolerance* (i.e., the dose needed to achieve satiation) over time depending on the prescribed dose. We assumed that between 8 and 16% of individuals prescribed an opioid will engage in misuse of an opioid (i.e., take more than the prescribed dose, seek out additional opioids) and between 2 and 14% of those prescribed an opioid will develop opioid dependence [42]. Once an individual has been exposed to opioids, the *desire* state variable will change over time as a function of *tolerance* and the current dose used. *Satiation,* another time-updated agent variable, reflects the level of satiation with MME consumed during the current time step and is bounded by 0 and 1. In summary, at every daily time step, agents will take MME (either prescribed PO, illicit PO, or heroin/fentanyl) according to their *desire*. Following use of opioids, the agent’s *tolerance*, *desire*, and *satiation* will be updated accordingly. Depending on the type of opioid used, there is a differential chance of overdose and death. Due to lack of peer-reviewed literature or trial data to inform these internal states, we used qualitative data from ethnographic and clinical research [43–45], anecdotal accounts and social media data to parameterize and then calibrated the model to reproduce overdose and mortality rates in North Carolina from 2010 to 2019.

### Treatment

Informed by the peer-reviewed literature and national surveys, we assume that a quarter of individuals who are currently misusing prescription opioids or using heroin would seek treatment, including non-pharmacologic treatment such as detoxification, at least once per year [27, 28]. Based on information from the National Survey of Substance Abuse Treatment Services (N-SSATS) for North Carolina, we assumed around 25,000 individuals currently receive treatment for OUD. For those receiving MOUD, agents are probabilistically assigned to different types of treatment (59% to methadone, 38% to buprenorphine-naloxone, and 3% to naltrexone). The probability of overdose and treatment retention varies by treatment modality [3, 30–32].

### Diverted buprenorphine networks

Buprenorphine diversion is defined as the unauthorized rerouting of prescription buprenorphine to an unintended recipient, either voluntarily or involuntarily, and with or without exchange of money or goods [46]. Within the ABM, we simulate diversion through the following steps: (1) An agent is inducted onto buprenorphine treatment for OUD, (2) the agent diverts a certain number of doses from their prescribed supply (typically 1 to 2 doses per week) to individuals with OUD within their peer network, (3) the agent receiving the diverted buprenorphine replaces usage of their typical opioid (i.e., heroin/fentanyl and/or non-medical use of a PO) with the buprenorphine on the day they receive the buprenorphine. We assume that users replace their use of opioids that day because of buprenorphine’s blocking or blunting effect of the euphoria of other opioids and that concurrent use of buprenorphine and a full or partial opioid agonist (e.g., heroin, PO) can result in precipitated withdrawal [47]. Agents seeking diverted buprenorphine will receive a single day’s dose from friends or peers or up to 3 days’ doses from a dealer. We do not simulate the exchange of money, goods, or services for diverted buprenorphine or the use of illicitly manufactured buprenorphine. We assume that on the day the diverted buprenorphine is consumed, agents have the same risk of overdose as agents taking prescribed buprenorphine. This parameter is varied within a sensitivity analysis (described below). We also assume that diverted buprenorphine is co-formulated with naloxone in the form of sublingual filmstrips or tablets as this formulation has become standard of care for treatment of OUD in preference to buprenorphine monotherapy in the USA except in specific circumstances such as during pregnancy and any injectable formulations are unlikely to be diverted [47].

We informed the probability of sharing or selling buprenorphine, conditional on having a current prescription, based on reports from Lofwall et al. [6] and Kenney et al. [36]. We assume that 10% of agents who misuse PO or use heroin/fentanyl will also use diverted buprenorphine [37–40]. For the 10% of agents with illicit PO or heroin use who also use diverted buprenorphine, we assume that the agent has a 15% daily probability of using diverted buprenorphine vs. heroin/fentanyl or PO [2]. We assume that 80% of diverted buprenorphine is sourced from a friend rather than a dealer [36, 41]. We incorporate assortative mixing within the model so that agents who have ever had a buprenorphine prescription are more likely to develop connections with other agents who have ever had a buprenorphine prescription or have used diverted buprenorphine. Due to the persistent stigma associated with the use of MOUD, we implemented this assortative mixing parameter to simulate the growth of networks of individuals who hold less stigma toward the use of buprenorphine [48]. Additionally, this can reflect friends introducing their network to friends who are prescribed and willing to share or sell buprenorphine. Individuals are assumed to share with 1 to 2 peers.

### Overdose

Agents can non-fatally or fatally overdose when consuming PO or heroin/fentanyl. The probability of overdose varies dependent on the dose (in MME) and whether the consumed opioid is heroin/fentanyl. Agents currently on MOUD have overdose probabilities as informed by the literature [3, 30]. The probability of dying conditional on experiencing overdose varies depending on whether naloxone is available.

### Sensitivity analyses

We varied the following in sensitivity analyses: (1) doubled the risk of overdose when using diverted buprenorphine to simulate concurrent consumption of benzodiazepines, alcohol, or other substances or use of diverted buprenorphine for euphoria; (2) increased the daily probability of using diverted buprenorphine from 15 to 25% for users of diverted buprenorphine; (3) increased the daily probability of using diverted buprenorphine from 15 to 50% for users of diverted buprenorphine; and (4) diverted 3% of the total buprenorphine diverted to be available to opioid-naïve agents (chosen at random) within the general population. The opioid-naïve agents with access to the diverted buprenorphine had a 1% probability of misusing the buprenorphine.

The lack of a defined toxic or lethal range for buprenorphine in postmortem blood along with a good safety profile complicates parameterizing the overdose probability for an opioid-naïve individual taking diverted buprenorphine [49]. Pediatric populations and individuals concurrently consuming central nervous system depressants (e.g., benzodiazepines, ethanol) are at the highest risk of overdose death [49]. Within this sensitivity analysis, we assumed that opioid-naïve individuals had a 0.3% probability of overdose death upon consumption of diverted buprenorphine but lack definitive data to inform this estimate. This is likely an overestimate. Of 27,275 buprenorphine oral exposures reported to US Poison Control Centers from 2003 to 2019, there were 84 fatalities (0.3%) including pediatric and intentional (e.g., suspected suicide) exposures [50].

We ran each model scenario 100 times with 10,000 agents. Output was scaled to reflect the full population of North Carolina (10.5 million residents). As a simulation study, this analysis was determined to not require review by RTI International’s Institutional Review Board. The model is programmed using NetLogo.

## Results

Over 5 years within the *status quo* scenario (i.e., low level of buprenorphine diversion, high levels of fentanyl within heroin supply), the model simulated 80,833 (90% credible interval [CI]: 77,736–84,016) opioid overdoses with 10,658 (CI: 9,699–11,679) fatal opioid overdoses (Table 2). On average, individuals sharing buprenorphine are connected to 5 (range 3–10) peers.

**Table 2:** Simulated outcomes for varying levels of buprenorphine diversion within the comprehensive opioid policy agent-based model

Model scenario	Total cumulative overdoses over 5 years mean (CI)^a^	Total cumulative fatal overdoses over 5 years mean (CI)^a^	Number and percent of averted fatal overdoses vs. status quo
*Main analysis*			
A. Status Quo (50% of those prescribed divert buprenorphine, 10% of individuals with OUD use diverted buprenorphine)	80,833 (77,736, 84,016)	10,658 (9699, 11,679)	n/a
B. Controlled prescription only (0% of those prescribed divert buprenorphine, 0% of individuals with OUD use diverted buprenorphine)	81,192 (78,241, 84,091)	10,741 (9895, 11,650)	+ 83 (0.78%)
C. Increased diversion (95% of those prescribed divert buprenorphine, 50% of individuals with OUD use diverted buprenorphine)	78,898 (76,087, 81,425)	10,301 (9439, 11,244)	− 357 (− 3.35%)
*Sensitivity 1: Increased overdose risk with diverted buprenorphine (from 2.08 to 4.16 per 100 p-y)*			
A. Status Quo	80,162 (77,310, 83,511)	10,536 (9557, 11,443)	n/a
B. Controlled prescription only^b^	81,192 (78,241, 84,091)	10,741 (9895, 11,650)	+ 205 (1.95%)
C. Increased diversion	77,924 (74,981, 80,634)	10,288 (9558, 11,149)	− 248 (− 2.35%)
*Sensitivity 2: increased probability of using diverted buprenorphine from 15 to 25%*			
A. Status Quo	80,564 (77,643, 83,014)	10,603 (9793, 11,458)	n/a
B. Controlled prescription only^b^	81,192 (78,241, 84,091)	10,741 (9895, 11,650)	+ 138 (1.30%)
C. Increased diversion	78,189 (75,313, 80,931)	10,288 (9436, 11,147)	− 315 (− 2.97%)
*Sensitivity 3: increased probability of using diverted buprenorphine from 15 to 50%*			
A. Status Quo	79,767 (77,018, 82,332)	10,475 (9582, 11,340)	n/a
B. Controlled prescription only^b^	81,192 (78,241, 84,091)	10,741 (9895, 11,650)	+ 266 (2.54%)
C. Increased diversion	75,372 (72,221, 78,788)	9897 (8999, 10,738)	− 578 (− 5.52%)
*Sensitivity 4: 3% of diverted buprenorphine available to opioid-naïve agents with a 1% probability of use and 0.3% fatal overdose probability*			
A. Status Quo	80,857 (77,932, 83,861)	10,666 (9931, 11,339)	n/a
B. Controlled prescription only^b^	81,192 (78,241, 84,091)	10,741 (9895, 11,650)	+ 75 (0.70%)
C. Increased diversion	79,163 (76,738, 81,804)	10,362 (9571, 11,120)	− 293 (− 2.75%)

*p-y*, person-years, *CI* credible interval

^a^Outcomes reported are means and 90% credible interval from 100 model runs

^b^Values are identical to that of the main analysis since parameters varied within the sensitivity relate to diverted buprenorphine

In a scenario where no buprenorphine diversion occurred (e.g., *controlled prescription only*), the total number of overdoses were similar with 83 additional fatal overdoses or an increase of 1% compared to the *status quo* scenario.

In a scenario where buprenorphine diversion occurred more frequently, there were 357 fewer fatal overdoses, with a decrease of 3.35% compared to the *status quo* scenario.

When the probability of overdose when using diverted buprenorphine was doubled from 2.08 to 4.16 per 100 person-years within a sensitivity analysis, both the *status quo* and *increased diversion* scenarios had fewer fatal overdoses compared to the scenario with no diversion.

As the probability of using diverted buprenorphine increased, the number and percent of averted fatal overdoses also increased. Up to 5.52% of fatal overdoses were averted in a scenario where agents had a 50% probability of using diverted buprenorphine and half of individuals with OUD used diverted buprenorphine.

In a sensitivity diverting buprenorphine to opioid-naïve individuals, there were 10,666 fatal overdoses or an increase of 8 (0.08%) compared to the *status quo* main scenario. In the *increased diversion* scenario, there were 10,362 fatal overdoses or 296 (2.86%) averted fatal overdoses compared to sensitivity *status quo*.

**Fig. 1:**
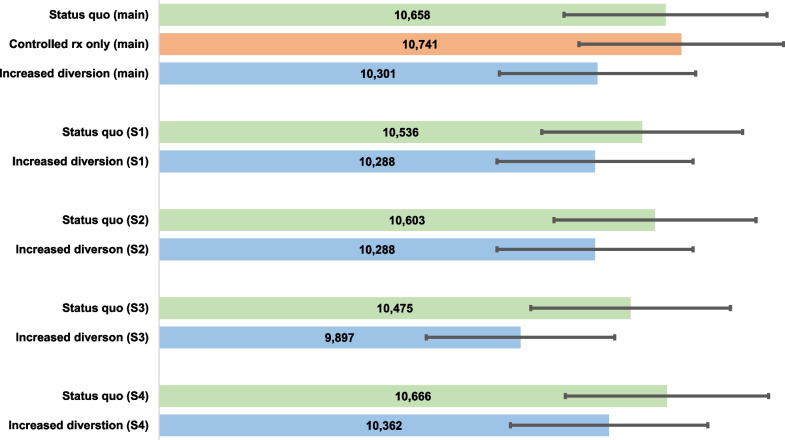
Cumulative number of fatal opioid overdoses over 5 years for model scenarios. S1—sensitivity 1 (increased overdose risk when using diverted buprenorphine from 2.08 to 4.16 per 100 person-years), S2—sensitivity 2 (increased daily probability of using diverted buprenorphine from 15 to 25% for users of diverted buprenorphine), S3—sensitivity 3 (increased probability of using diverted buprenorphine from 15 to 50%), S4—sensitivity 4 (3% of diverted buprenorphine available to opioid-naïve agents with a 1% probability of use and 0.3% fatal overdose probability).

Across all scenarios and sensitivities, the *controlled prescription only* scenario remained the scenario with the highest number of overdoses (Fig. 1); however, the credible intervals for the modeled scenarios overlap.

## Discussion

Up to an estimated 87% of people with OUD do not receive evidence-based treatment [51]. Lack of access has driven individuals to procure MOUD outside of formal channels, including the acquisition of diverted buprenorphine [12]. Recognizing that substantial barriers to treatment and an unmet need for MOUD-based treatment likely drives buprenorphine diversion, several states have decriminalized buprenorphine possession [52]. Decriminalization of buprenorphine possession, increased flexibility around buprenorphine prescription, and the removal of the X-waiver will likely increase buprenorphine diversion as an unintended effect. In this simulation study, we used an agent-based model as a policy tool to estimate the impact of diverted buprenorphine on statewide incidence of opioid overdose. We found that overdoses were similar or decreased, rather than increased, with policies that could lead to greater diversion of buprenorphine.

Our results support existing literature that the use of diverted buprenorphine will lower the risk of overdose if it is replacing the use of illicit substances with a high likelihood of fentanyl contamination [11]. Carlson et al. interviewed 356 individuals who reported use of diverted buprenorphine in the past 6 months and found that use of diverted buprenorphine was protective and that increased frequency of use was associated with a greater reduction in the odds of overdose [2]. Even when doubling the probability of overdose for users of diverted buprenorphine, simulated scenarios with buprenorphine diversion had lower likelihood of opioid overdose and overdose deaths compared to a scenario with no diversion. On average, these scenarios had fewer fatal overdoses compared to the *status quo* and *increased diversion* scenarios within the main analysis; however, these small differences were likely driven by the stochasticity of the model and do not represent a true difference.

Fears have been expressed regarding the initiation of opioid-naïve individuals into opioid use through use of diverted buprenorphine [53]. When France lowered restrictions related to accessing buprenorphine, the country experienced a moderate increase in those reporting misuse of buprenorphine, specifically with single-formulation buprenorphine (vs. preparations co-formulated with naloxone) [54]. However, the overall overdose rate fell precipitously and is currently a fraction of the US rate [54]. The reports of buprenorphine misuse in France were primarily among individuals with OUD and a history of injection drug use rather than individuals initiating opioid use [54]. There is little evidence to suggest the uptake of diverted buprenorphine by opioid-naive individuals is occurring within the USA [11, 13]. Accordingly, our main analysis did not simulate this pathway to opioid use. However, to quantity the potential impact of use of diverted buprenorphine by opioid-naïve individuals, we conducted a sensitivity analysis where 3% of the diverted buprenorphine is available to opioid-naïve agents. When accounting for the potential diversion of buprenorphine to opioid-naïve agents, positive impacts of diverted buprenorphine were slightly attenuated. Notably, the overdose risk of buprenorphine use among opioid-naive individuals is likely low as indicated in data showing that even with significant increases in buprenorphine prescription during the COVID-19 pandemic, there were no concomitant increase in the proportion of overdoses attributable to buprenorphine [1]. While there was no reported increase in overdoses, a 65-fold increase in buprenorphine oral exposures reported to US Poison Control Centers occurred nationally from 2003 to 2019 [50]. Therefore, surveillance on the accidental use of diverted buprenorphine or use of buprenorphine for euphoria is warranted while noting that the use of diverted buprenorphine is less likely to result in overdose compared to other prescription opioids and much less likely than with illicit opioids such as heroin or fentanyl for opioid-naïve as well as opioid-experienced individuals [2].

Opioid use debut with buprenorphine in opioid-naïve individuals appears to occur infrequently in the USA. In a study using national data from 2013 to 2016, Rege et al. report that only 6% of pediatric buprenorphine poison exposures among youths aged 19 or younger were due to the euphoric effect of the drug [9]. Lavonas et al. report that rates of misuse for buprenorphine were highest for single-formulation pills but these rates were much lower compared to that of other prescription opioids [10]. Structured surveillance of Internet forums found that people perceive buprenorphine/naloxone film strips as “weak” from a euphoria perspective [10]. Importantly, lessening restrictions does not obviate the need for patient education on safe storage practices to reduce the risk of accidental exposure for children and/or intentional misuse by opioid-naïve individuals [9, 10].

Diverted buprenorphine is primarily shared through peer networks rather than purchased from a dealer [36, 41]. Qualitative work has reported that distributing buprenorphine among peers, particularly to an individual in withdrawal, has altruistic motivations [55]. This is particularly evident among couples where only one partner is prescribed an MOUD [55]. However, the desire to sell buprenorphine for profit and as a business venture has also been reported [55]. Many of those purchasing diverted buprenorphine would likely prefer a prescription which could cost less and is more reliable [11]. Use of diverted buprenorphine may prepare individuals for a smoother transition into formal treatment as individuals are already familiar with the medication and have demonstrated belief in the medication’s efficacy. In at least one observational study, the use of diverted buprenorphine prior to treatment was associated with improved retention and decreased illicit opioid use once linked to treatment [56]. Use of diverted buprenorphine can be an indicator that an individual is ready for treatment. On the other hand, qualitative work has shown that experiences of precipitated withdrawal while using diverted buprenorphine might make individuals resistant to starting formal buprenorphine treatment [57, 58]. Several participants report that experiencing precipitated withdrawal from fentanyl-adulterated heroin has made them reluctant to use buprenorphine, diverted or through formal channels, for OUD treatment [58]. In addition, participants who used diverted buprenorphine perceived formal treatment as cumbersome, stigmatizing, and unreliable and viewed use of diverted buprenorphine as granting them autonomy [57]. Sharing the benefits of engaging in formal treatment such as close management of buprenorphine induction to prevent symptoms of precipitated withdrawal as well as access to other medical care or services such as housing and food assistance could potentially help individuals link to care. There is a need for additional research to understand how use of diverted buprenorphine impacts uptake of formal OUD treatment and ways to address patient concerns over autonomy, stigma, and accessibility.

This analysis is subject to several limitations. This model is calibrated and parameterized to reflect a generic North Carolina setting using statewide averages for treatment access, overdose rates, and other parameters. Future work is planned to compare differences in the impact of buprenorphine diversion by urbanicity (i.e., rural vs. urban settings) and by varying levels of treatment access. Due to a lack of available data, national studies were primarily used to inform diverted buprenorphine networks. Internal agent states related to opioid use (e.g., tolerance, satiation, desire) were calibrated to reproduce North Carolina’s trends in opioid-related mortality due to lack of peer-reviewed literature to inform these parameters. Accidental ingestion of fentanyl through non-opioid sources (e.g., stimulant use) was not explicitly simulated. A further limitation is that we are not able to account for all the factors influencing opioid use and overdose such as poverty and barriers to treatment access within this simulation study.

## Conclusions

The movement to decriminalize buprenorphine supports efforts to provide a safer alternative to heroin or fentanyl for individuals who misuse opioids. An additional step would be to lower the existing barriers to MOUD so that anyone seeking treatment is able to access care. Policy and provider-level barriers to accessing buprenorphine include a shortage of buprenorphine providers and pharmacies carrying buprenorphine, stigma against persons with OUD and/or use of MOUD, inadequate prescriber reimbursement, and the perceived difficulty of managing treatment [6, 7]. While significant progress has been made in addressing these barriers through actions like eliminating the X-waiver and increased funding of loan repayment programs for MOUD prescribers, there is more work to be done. Unfortunately, patient-level barriers continue to challenge MOUD uptake. Individuals with OUD frequently face barriers when navigating the healthcare system, socioeconomic challenges such as unstable housing and poverty, and comorbidities such as mental health disorders and chronic pain [6, 7]. Harm reduction-based policies should focus on reducing barriers to accessing and continuing MOUD-based treatment rather than restricting access to buprenorphine to prevent diversion. Liberalizing buprenorphine prescription policies is predicted to increase the diversion of buprenorphine. This modeling study found that increased buprenorphine diversion is unlikely to increase opioid-related overdose [2, 3].

### Supplementary Information

**Additional file 1**. Supplemental File summarizing key parameters, sources, model processes and assumptions.

## Data Availability

The datasets used and/or analyzed during the current study will be made available from the corresponding author on reasonable request.

## Abbreviations

ABM: Agent-based model
CI: Credible interval
COVID-19: Coronavirus disease 2019
FDA: Food and Drug Administration
MME: Morphine milligram equivalent
MOUD: Medications for opioid use disorder
NIDA: National Institute on Drug Abuse
N-SSATS: National Survey of Substance Abuse Treatment Services
OUD: Opioid use disorder
PO: Prescription opioid
p-y: Person-years
