# Supplementary material for: Examining buprenorphine diversion through a harm reduction lens: an agent-based modeling study
Source: Harm Reduct J. 2023 Oct 17;20:150. doi: 10.1186/s12954-023-00888-6 (PMC10580611; doi:10.1186/s12954-023-00888-6)
Supplement: Supplementary file 1 — Additional file 1. Supplemental File summarizing key parameters, sources, model processes and assumptions. [file 12954_2023_888_MOESM1_ESM.pdf]

SUPPLEMENTAL MATERIAL FOR:

Examining buprenorphine diversion through a harm reduction lens: An agent-based modeling study

Joëlla W. Adams, MPH, PhD*, Michael Duprey, BA*, Sazid Khan, PhD*,

Jessica Cance, MPH, PhD*, Donald P. Rice, MD†, Georgiy Bobashev, MS, PhD*

* RTI International, Research Triangle, NC, USA

† Division of Infectious Disease, Department of Medicine, Alpert Medical School of Brown University, Providence, RI, USA

Updated: 09/12/2023

12 Tables, 2 Figures

Table of Contents

Introduction…… 3
Purpose…… 4
Entities, State Variables, and Scales…… 5
Process Overview and Scheduling…… 8
Model Initialization…… 10
Acute and Chronic Pain Opioid Prescriptions…… 11
Opioid Use States…… 18
Agent Networks…… 20
Treatment for Opioid Use Disorder…… 23
Overdose…… 25
Model Calibration…… 27
References…… 28

Introduction

In North Carolina, overdose-related deaths have continued to grow over the last several years, with over 4,000 deaths in 2021 (https://www.ncdhhs.gov/opioid-and-substance-use-action-plan-data-dashboard). Efforts to liberalize the prescription of buprenorphine-naloxone (buprenorphine) for the treatment of opioid use disorder is part of the national strategy to decrease opioid overdose deaths. However, policymakers have expressed concerns about these efforts leading to the diversion of buprenorphine. However, it is likely that the harm-reduction benefit of diverted buprenorphine is greater than the risks associated with diversion as diverted buprenorphine could be used in place of heroin or fentanyl. The model herein described allows for investigation of buprenorphine diversion and its impact on fatalities and overdoses in North Carolina.

This supplement provides detailed information on modeling processes, parameter sources, and assumptions related to this analysis. The ABM incorporates a social network structure and transition matrix with agent interactions including dealer-user relationships, user networks, and access of medication through emergency departments, pharmacies, and other healthcare settings. This model is intended for use in examining long-term effects of prevention and treatment interventions on opioid misuse. This supplement accompanies a manuscript detailing an analysis simulating networks of buprenorphine diversion to estimate the impact of diversion on overdose rates over five years. Details on the model scenarios, analytic overview, sensitivity analyses, and output for the specific analysis are included within the manuscript.

Purpose

This model evaluates the effects of diverted buprenorphine on opioid overdose and mortality in North Carolina. We consider the use of diverted buprenorphine in place of heroin or counterfeit pills. As a partial opioid agonist, buprenorphine has a superior safety profile compared to full *mu*-receptor opioid agonists such as methadone and oxycodone with respect to respiratory depression and fatal overdose.[1] Therefore, *a priori*, it is unclear if increased buprenorphine diversion will lead to increases in overdose deaths. Within this simulation, we estimate the 5-year effect of strategies that would increase the likelihood of buprenorphine diversion on opioid-related overdose and mortality.

Entities, State Variables, and Scales

The ABM has two types of entities—people and locations. People are referred to as “agents” and represent North Carolina residents over the age of 18. For this analysis, we modeled 10,000 agents to represent the 10.5 million residents of North Carolina. Locations include emergency departments, pharmacies, physicians, and dealers that represent physical locations where agents can receive prescribed opioids (PO), PO prescriptions, or obtain illicit PO and/or heroin.

People/Agents

Agents represent North Carolinians accessing healthcare for PO to treat acute and chronic pain, patients using MOUDS to treat opioid use disorder (OUD), or individuals misusing PO or using heroin. These agents may experience pain, uptake opioids, engage with the medical system, seek drugs from other people, and engage with drug dealers. Agents have many attributes (summarized in **Table S1**) that are both time invariant (e.g., primary pharmacy) and time varying (opioid *use-state*). Among these are variables to track prescription details, current drug supplies, as well as the agent’s internal state. The state variables and sources used to inform these parameters are described in more detail within the subsections titled “Acute and Chronic Opioid Prescriptions”, “Opioid use”, “Treatment for opioid use disorder”, “Agent Networks”, and “Overdose”.

Table S1. People state variables.

**Variable**	**Description**	**Time-varying**
Use-state	Opioid use state (categorical: prescription compliant, prescription noncompliant, prescription opioid use disorder, heroin use, heroin use disorder, cessation of opioid use)	Yes
Treatment-type	Type of treatment for opioid use disorder that person is currently receiving (categorical: residential/non-MOUD based, buprenorphine, methadone, naltrexone)	Yes
ever-diverted-bupe	Binary variable indicating if agent has ever diverted buprenorphine (yes/no)	Yes
using-div-bupe	Binary variable indicating if the agent is currently using diverted buprenorphine (yes/no)	Yes
PW_div_bupe	Binary variable indicating if the agent experienced precipitated withdrawal while using diverted buprenorphine (yes/no)	Yes
Pain-type	Type of pain experienced (categorical: none, chronic, acute)	Yes
Prescribed-dose	Most recent prescribed dose in MME (numerical, range: 10-unbounded)	Yes
Required-dose-for-pain	Dose needed to treat current pain as determined by *pain-type* and *desire* variables, (numerical, range: 10-unbounded)	Yes
PDMP_record	Totality of available prescribed opioids in MME (numerical)	Yes
Prescription-length	Length of most recent prescription in days (numerical)	Yes
Prescribers-Rx-list	List of previous physicians and prescribed doses	Yes
My_physicians	List of all physicians in model sorted by Euclidean distance from agent	No
My_pharmacy	Agent’s pharmacy	No
My_dealer	Agent’s primary dealer	Yes
Opioid-supply	Opioids in possession in MME (numerical)	Yes
Heroin-supply	Heroin in possession in MME (numerical)	Yes
Desire	Current desired amount of opioids in MME (numerical)	Yes
Tolerance	Current tolerance of opioids in MME (numerical)	Yes

Satiation	Current level of satiation (numerical, bounded by 0 and 1)	Yes

Locations

At each time step (fixed at 1 day), agents can interact with a location. Locations represent emergency departments, pharmacies, physicians, and dealers (**Table S2**). Throughout the model run, agents interact with locations which impact the agent's state variables (e.g., agent picks up a prescription from a pharmacy and the agent's *desire* for an opioid is fulfilled).

Table S2. Location type, number of nodes, and state variables in the ABM

Location Type	Description	Value	Source
Emergency Department	Physical location where people can pick up PO representing the number of emergency departments in NC	105 nodes	Centers for Medicare and Medicaid (https://data.cms.gov/provider-data/topics/hospitals)
Pharmacy	Physical location where people can pick up PO representing the number of pharmacies in NC	3,513 nodes	North Carolina Board of Pharmacy (http://www.ncbop.org/about/statistics.htm)
Physician	Entities that prescribe PO including physicians (primary care physicians, addiction specialists, surgeons), physician assistants, nurse practitioners, and dentists.	69,348 nodes	North Carolina Medical Board, American Dental Association, North Carolina Board of Nursing
Dealers	Agents who sell illicit PO and/or heroin/fentanyl	1.4% of total adult population	National Survey on Drug Use and Health (NSDUH), 2019

Emergency Departments

The "emergency department" location type represents a physical location where agents can pick up PO. Emergency departments can distribute opioids without including a pharmacy. We estimated the number of nodes by determining the number of hospitals in North Carolina with an emergency department as of September 2021. This was cross-referenced with information from the American Hospital Directory. This number does not include free-standing urgent care centers where agents may obtain prescription opioids.

Pharmacies

The "pharmacy" location type is another physical location where agents can pick up a PO. Pharmacies fill prescriptions written by physicians. This is the sole action of the "pharmacy" entity. We estimated the number of pharmacies using data provided by the North Carolina Board of Pharmacies which conducts a census survey done annually. Data is current as of 2020. We included both in-state pharmacies (n=2,722) and out-of-state pharmacies (n=791) that obtained licensure to send prescriptions to individuals living in North Carolina for a total of 3,513 pharmacy locations.

Physicians

The "physician" location type encompasses entities eligible to prescribe opioids based on the number of licensed physicians (MD and DO degree), physician assistants, nurse practitioners, and dentists in North Carolina. The number of physicians, physician assistants, and residents in training licensed in NC was sourced from the North Carolina Medical Board with data as recent as December 31, 2020. The number of dentists was reported by the American Dental

Association with data as recent as May 2021. The number of nurse practitioners was based on data from the North Carolina Board of Nursing with data from November 2021.

Dealers

The “dealer” location type includes entities that can sell illicit PO or heroin. We estimated the number of agents who sell prescription opioids or heroin illicitly based on the 2019 National Survey of Drug Use and Health. Within the survey, respondents self-report selling illegal drugs and we used a weighted national estimate to inform the number of dealers.

Temporal and Spatial Resolution and Scales

The ABM is implemented with a fixed single day time step. A 1-day time step was selected based on the assumption that an agent is primarily at one location each day. The number of daily time steps in the model is set by an input parameter. This analysis used a 5-year time horizon.

Although the model can represent agents and locations geospatially, we use an abstract 2D-space that is not representative of the spatial properties of any real location. Rather than using physical distance, we consider a list of potential pharmacies and emergency departments. However, in selection of a primary care physician, the closest (in Euclidian space) physician is elected. Other physicians of choice are recorded as lists.

Process Overview and Scheduling

At each timestep, a person who uses opioids checks their available supply of opioids and, depending on their desire, chooses to use some or no portion of their supply. A person who is not using opioids can be in pain (acute or chronic) and can obtain a prescription for an opioid. A physician can prescribe or not prescribe opioids and chooses the dose depending on the need and regulations. Emergency departments can also distribute opioids to the patients depending on regulations. Each prescription is supposed to be written to Prescription Drug Monitoring Program (PDMP) system and the decision on whether to prescribe or not to prescribe medication can be made based on this record depending on a physician's compliance with the regulations. Dealers wait for a person who uses opioids to approach them for a desired quantity of drugs and will attempt to provide them with that dose. The dose can be a fatal amount.

Agents are connected into networks by having lists of their connections. For example, a patient has a list of physicians to go to for a new prescription. In the same way, a person who is misusing opioids has a list of known dealers. Additionally, agents are connected in "social" networks which could be used to share drugs.

The model was implemented in NetLogo and commands are executed asynchronously. Upon model initialization, agents are assigned an arbitrary unique identifier. Loops acting over all of an agent type (e.g. **for** all dealers **do**) act on agents sequentially with respect to their unique identifier. **Figure S1** summarizes the model's executed processes. The **go** procedure is called at each timestep of the simulation.

The details of each of the process are complex and rather than following design items in the ODD protocol, we described processes and agent's behaviors in detail in the Appendix B following model initialization.

Figure S1. Process scheduling for Comprehensive Opioid Policy Agent-based Model

Algorithm 1 GO

```
procedure GO
    if time mod 30 = 0 then
        for all people do
            PDMP-record ← 0                                  ▷ Resets PDMP record every 30 days
        end for
        for all dealers do                                   ▷ Restock dealers' drug supplies
            opioid-supply ← max-opioid-supply
            if RandFloat(1) < bad-batch-probability then
                bad-heroin-supply ← max-heroin-supply
                heroin-supply ← 0
            else
                heroin-supply ← max-heroin-supply
                bad-heroin-supply ← 0
            end if
        end for
    end if
    for all people do
        SWITCH-USE-STATES                                    ▷ Run the SWITCH-USE-STATES submodel
        if use-state = Rx-comp then
            RX-COMP                                          ▷ Run the RX-COMP submodel
        end if
        if use-state ∈ {Rx-noncomp, POUD, HU, HUD} then
            RX-NONCOMP                                       ▷ Run the RX-NONCOMP submodel
        end if
        if use-state = quit then
            QUIT                                             ▷ Run the QUIT submodel
        end if
    end for
end procedure
```

Model Initialization

The model is currently parametrized to reflect conditions in North Carolina from January 2010 to December 2019. Certain parameters were deliberately selected before the COVID-19 pandemic as the pandemic lead to temporary changes in treatment access. We have noted where this is the case. Data from the North Carolina Department of Health and Human Services (NCDHHS) on the statewide rate of fatal overdose was used to calibrate the model. Parameters linked to sources that change over time were taken from those sources as of the specified start date. Initializing values for specific parameters are described within the below subsections.

Appendix B. Details of the model processes.

Acute and Chronic Pain Opioid Prescriptions

Within this subsection, we describe how acute and chronic pain are simulated within the model and the process through which people seek and acquire prescription opioid medication to treat pain. Agents are trifurcated by *pain-type*: none, acute, and chronic. Differences in *pain-type* impact the quantity of opioids needed to treat the underlying pain and the length of treatment needed, both internally and as estimated by a physician.

Acute pain

Acute pain is defined as pain lasting for less than three months. Examples of opioid prescription for acute pain include prescriptions following surgery, dental procedures, or physical trauma. Parameters related to acute pain are summarized in **Table S3**.

To estimate the number of individuals in North Carolina who experience acute pain, we used a retrospective cross-sectional analysis by Mikosz et al. from 2016 to 2017 with 18,016,259 patients with private insurance and 11,453,392 Medicaid enrollees.[2] During this time period, there were 3,686,310 visits for nonsurgical acute pain (12.5% over one year) and 671,250 surgical procedures (2.27% over one year). Therefore, we estimated that around 14.79% (0.125+0.227) of the total North Carolina population experience an acute pain episode over one year. As reported by Mikosz et al., there were 3,686,310 visits for nonsurgical acute pain where 13-16% of patients were prescribed an opioid and 671,250 surgical procedures where between 55-65% of patients were prescribed an opioid.[2] Therefore, we assumed that 15% of nonsurgical visits resulted in an opioid prescription (n=552,947) and 60% of surgical visits (n=402,750) for a total of 955,697 of 4,357,560 (22%) visits resulting in an opioid prescription. On average, we assume that 22% of visits for acute pain would result in an opioid prescription.

We based the amount of opioids needed to treat acute pain on an a population-based analysis by Howard et al. which examined the quantity of opioids prescribed following surgery for patients over the age of 18 (n=2392) across 33 health systems in Michigan in 2017.[3] They reported the opioids prescribed and the opioids consumed in oral morphine equivalents (OME). Median prescribed OME was 150 (IQR 135-225). Median consumed OME was 45 OME (IQR 5-125) meaning that median consumption was 27% of the prescribed amount and 24% of patients took no opioids after surgery. Based on the average prescription length, this results in a median of 6 (1-17) morphine equivalents per day. There is likely a large variance in underlying pain for patients offered an opioid prescription to treat acute pain. This is also informed by the probability of an individual seeking an additional prescription to treat acute pain (which ranged from 12-30%). To model this, we assumed that 70% of patients prescribed an opioid for acute pain had their pain sufficiently treated. The other 30% had underlying pain that did not get sufficiently alleviated and would seek additional pain medication. The amount of MME initially prescribed for acute pain was informed by Mikosz et al.[2] They report the mean daily opioid dosage was relatively constant across nonsurgical acute pain conditions- approximately 30 MME/day. Dosages prescribed for postsurgical pain ranged more widely from 37.4 MME for partial mastectomies to 64 MME for combined spinal fusion procedures. However, postsurgical pain represented only 15% of total acute pain visits. Therefore, we implemented 30 MME/day for acute pain with a range to reflect variation due to condition or postsurgical procedure.

The mean length of opioid prescription for acute pain of 7 days was based on data reported by Mundkur et al. and Dowell et al.[4,5] The implemented distribution (3 to 7 days) was based on CDC recommendations from 2016 which state that "when opioids are used for acute pain,

clinicians should prescribe the lowest effective dose of immediate-release opioids and should prescribe no greater quantity than needed for the expected duration of pain severe enough to require opioids. Three days or less will often be sufficient; more than seven days will rarely be needed".[4] For acute pain, Mundkur et al. reports that the probability of a refill after an initial 7-day prescription ranged from 12% to 30%, depending on the condition with a refill probability of less than 25% for most conditions.[5]

Table S3. Parameters related to acute pain.

Parameter or Variable	Description	Value, distribution	Source
Probability of an agent developing acute pain	Probability of an agent developing acute pain over 12 months	15% (n=1,211,171 in North Carolina)	Mikosz et al.[2]
Proportion of agents with acute pain with an opioid prescription	Percent of agents presenting with an acute condition (post surgical or physical trauma) who receive a prescription opioid	22%	Mikosz et al.[2]
Mean-acute-pain	The initial daily mean MME needed in order to treat acute pain (e.g., underlying pain level)	6 MME for 70%, 30 MME for 30%	Howard et al.[3]
Mean-acute-pain-Rx	The initial daily mean MME prescription picked up at pharmacy for acute pain	30 MME (normal, 10 SD)	Mikosz et al.[2]
Prescription-length	Length of prescription in days for acute pain	7 days (3-7 days)	Mundkur et al.[5], Dowell et al.[4]
Acute-mean-prescribing-tendency	Probability that an individual physician refills a prescription opioid or increases dosage of an opioid to a patient reporting acute pain after initial prescription	25% (uniform, 12-30%)	Mundkur et al.[5]

Chronic pain

Chronic pain is defined as pain lasting for more than three months. Parameters related to chronic pain are summarized in **Table S4**. The U.S. Census reported that there were 8,191,000 individuals over the age of 18 in North Carolina as of July 1, 2019. Using data from the 2019 National Health Interview Survey, Zelaya et al. estimate that 20.4% of U.S. adults had chronic pain in the past three months.[6] Therefore, 1,671,000 (20.4%*8,191,000) adults in North Carolina are estimated to experience chronic pain. We based the proportion of agents with chronic pain receiving an opioid prescription on a 2020 systematic review and meta-analysis which aggregated data from 42 studies with 5,059,098 patients, mostly based in the United States, which found that 30% of patients with chronic, non-cancer pain were prescribed an opioid.[7]

To capture the underlying mean MME needed to treat chronic pain or the “desired" dose for agents with chronic pain, we assumed that the initial dose needed is 52 mg/day. This was informed by a randomized pragmatic randomized clinical trial with 135 Veterans referred to a specialty pain clinic who were followed for 12 months to compare the effectiveness of a conservative “hold the line” approach to prescribing opioids compared to a more liberal dose escalation strategy. At the end of the trial, the mean MED was 52 mg/day with the liberal approach compared to 40 mg/day with the maintenance approach. The initial opioid prescription dosage (50 MME) and length (7-28 days) for chronic, non-cancer pain was informed by 2016 CDC guidelines.[4] The guidelines state that “clinicians should evaluate benefits and harms with patients within 1 to 4 weeks of starting opioid therapy for chronic pain or of dose escalation. Clinicians should evaluate benefits and harms of continued therapy with patients every 3

months or more frequently. If benefits do not outweigh harms of continued opioid therapy, clinicians should optimize other therapies and work with patients to taper opioids to lower dosages or to taper and discontinue opioids." However, suddenly tapering patients with chronic pain and opioid prescriptions could have unintended consequences, including individuals seeking out illicit opioids or heroin with concomitant increased risk of overdose. In response, Health and Human Services issued guidelines in 2019 that urged physicians to work with individual patients on lowering dosage or tapering use. Due to substantial changes over time and the potential risks of suddenly discontinuing an opioid for chronic pain for a non-naive patient, we assume that a high percentage (90%) of physicians would re-fill an existing prescription.

Table S4. Parameters related to chronic pain.

Parameter or Variable	Description	Value, distribution	Source
Number of agents experiencing chronic pain	Agents experiencing chronic pain (with or without opioid use). Estimate specific for NC.	1,671,000	Zelaya et al.[6]
Proportion of agents with chronic pain with an opioid prescription	Percent of agents presenting with chronic pain who receive a prescription opioid	30%	Mathieson et al.[7]
Mean-chronic-pain	The initial daily mean MME needed in order to treat chronic pain (e.g., underlying pain level)	52 MME (normal, 10 SD)	Naliboff et al.[8]
Mean-chronic-pain-Rx	The initial daily mean MME prescription picked up at pharmacy for chronic pain	50 MME (normal, 10 SD)	Dowell et al.[4]
Chronic-prescription-length	Length of prescription in days for chronic pain	28 days (7-28 days)	Dowell et al.[4]
Chronic-mean-prescribing-tendency	Probability that an individual physician refills a prescription opioid or increases dosage of an opioid to a patient reporting chronic pain after initial prescription	90%	Dowell et al.[4] , HHS[9], FDA[10]

Opioid prescriptions to treat acute or chronic pain

All patients in the model who suffer from acute or chronic pain have a probability of seeking treatment from a physician or an emergency department.

Emergency departments

Emergency department state variables (**Table S5**) include the probability of distributing opioids to a patient and compliance with CDC recommended limits on opioid prescriptions. An agent accessing an ED for pain will select an arbitrary ED from the set of all EDs and visit. In this visit, the ED first probabilistically determines whether they will prescribe the agent opioids during this visit, as determined by the prescribing tendency. Before finalizing the prescription/distributing opioids to the agent, the ED completes two additional checks: a dose-cap check and a PDMP check. Both checks are probabilistically determined, governed by the dose-cap-compliance and PDMP-compliance parameters associated with the particular ED. If a dose-cap check is performed, if an agent is to be assigned a prescription > 90 MME/day, the prescription is instead capped at 90 MME.

Similarly, if a PDMP check is performed, the ED queries the PDMP. If the PDMP record for the given agent shows a combined prescription > 90 MME/day, no new prescription is written by the ED. If, however, a prescription is written for this current visit, the PDMP record is updated to

reflect the new prescription written. Finally, the agent's supply of opioids are updated to reflect any distributed opioids.

Prescription tendencies for emergency departments were informed by the literature. Barnett et al. performed a retrospective analysis involving Medicare beneficiaries (n=215,678) who had an index emergency department visit from 2008-2011 and were opioid-naïve (no prescription for opioids in the previous six months).[11] They found that the overall rate of opioid prescribing for patients reporting an injury (n=37,083) was 23.7% but varied by provider (low-intensity providers prescribed an opioid 12.6% compared to high-intensity providers [35.8%]).[11] Therefore, we implemented a 23.7% probability with a uniform distribution from 12.6-35.8%. Borrelli et al. conducted a cross sectional observational study using data from the RI Prescription Drug Monitoring Program on controlled substance prescriptions dispensed in 2018 and found that 75% were compliant with PDMP laws.[12] Hung et al. report that from 2017 to 2018, 20% of NC Medicaid enrollees were found to have been prescribed overlapping opioid and non-opioids which indicated a lack of checking the PDMP.[13] Therefore, we assume that between 75-80% of physicians are compliant with PDMP laws in North Carolina.

Initial dose was also informed by the literature. An analysis of electronic health records from 2015 to 2017 (n=8,652) sought to determine if the release of 2016 CDC guidelines on opioid prescriptions was associated with changes in prescribing habits within the emergency department of an academic medical center.[14] The average MME decreased from 30.6 (SD=20.2) pre-guidelines to 29.8 (SD: 19.5) post-guidelines. To further validate, we compared this estimate to the average dispended MMEs reported by Sun et al. who examined 1,187,237 ED visits and reported an average of 110 MME (SD=115) prescribed. If we assume that the days prescribed ranged from 3-5, this would be 22-36.7 MME which is in line with the reported MME from Dayer et al.[15]

Table S5. State variables for emergency departments

Variable	Description	Value, distribution	Source
Prescribing-tendency	Probability that an individual emergency department prescribes an opioid or increases dosage of an opioid to a patient reporting pain	23.7% (uniform: 12.6-35.8%)	Barnett et al.[11]
Dose-cap-compliance	Percentage of prescriptions complying with cap on dosage (<90 MME/day)	92.7%	Centers for Disease Control and Prevention. 2019 Annual Surveillance Report of Drug-Related Risks and Outcomes — United States Surveillance Special Report.[16]
PDMP-compliance	Percentage of physicians complying with prescription drug monitoring laws	77.5% (75-80%)	Borrelli et al.[12], Hung et al.[13]
Mean-ED-Rx	The initial mean MME prescription from an emergency department visit	29.8	Dayer et al.[14], Sun et al.[15]

Physicians

Physicians can write prescriptions for opioids, titrate up a patient's dose, and adhere to CDC's guidelines for maximum dose (**Table S6**). An agent will visit a physician and report chronic or acute pain. If a returning patient, the physician looks up their current (i.e., last written) prescription. If the patient reports to still be in pain with the current prescription, the physician

may up-titrate the dose. We assume that if the physician titrates up, they will do so by increasing the MME dose by 25% based on a report by Gallagher et al.[17] Limited information exists for how physicians would increase or titrate opioid dosages in response to continued pain reported by a patient considering newer understanding of how high doses of opioids increase the risk of OUD and overdose. To inform our parameter estimate, we used a case report from 2007 which describes how the recommended approach to titrating an opioid dose for a patient reporting unmanaged pain would be to increase the dose by 25%. Acute pain patients and chronic pain patients are prescribed opioids with differing probabilities, as described earlier.

If a new patient, the physician probabilistically determines whether they will prescribe the agent opioids during this visit, as determined by parameters described earlier. If the physician does prescribe opioids, they do so by sampling the amount from a distribution built around parameters related to dose. Before finalizing the prescription/distributing opioids to the agent, the physician probabilistically completes two additional checks: a dose-cap check and a PDMP check. Both checks are probabilistically determined, governed by the *dose-cap-compliance* and *PDMP-compliance* parameters associated with the physician. We assumed that "emergency departments" and "physicians" have the same value for compliance to prescription monitoring programs and maximum dosage caps recommended by the CDC. Finally, the physician updates their internal records to reflect any new prescriptions written for this patient.

Table S6. State variables for physicians

**Variable**	**Description**	**Value, distribution**	**Source**
Dose-cap-compliance	Percentage of prescriptions complying with cap on dosage (<90 MME/day)	92.7%	Centers for Disease Control and Prevention. 2019 Annual Surveillance Report of Drug-Related Risks and Outcomes — United States Surveillance Special Report[16]
PDMP-compliance	Percentage of physicians complying with prescription drug monitoring laws	77.5% (75-80%)	Borrelli et al.[12], Hung et al.[13]
acute-mean-prescribing-tendency<br><br>*chronic-mean-prescribing-tendency*	Probability that an individual physician refills an existing opioid prescription or increases dosage of an opioid to a patient reporting continued acute or chronic pain	Acute pain: mean 25%, SD 10%<br>Chronic pain: mean 90% SD 10%	Dowell et al.[4], Mundkur et al.[5]
Titrate-up-Rx	Mean percent increase in prescribed dose if patient reports continued pain	25%	Gallagher et al.[17]

Physicians cannot distribute opioids, only pharmacies can distribute opioids with a physician's prescription. If prescribed opioids by a physician, a patient will go to a pharmacy to fill the prescription and take the opioids over the course of a month.

Desire, tolerance, and satiation

We used ethnographic research, qualitative neurobiological descriptions and past model descriptions[18-23] to model the key internal components affecting the potential increase in

dosage. Three internal state model parameters (*desire, tolerance,* and *satiation*) apply to all agents in the model and change over time to determine the amount of MME used by agents (**Table S7**). Due to lack of peer-reviewed literature or trial data to inform parameterization, we used qualitative data from ethnographic and clinical research, anecdotal accounts and social media data to inform parameterization and then calibrated the model to reproduce overdose and mortality rates in North Carolina from 2010 to 2019. Calibration details are presented in a separate section below.

Desire reflects the amount of MME sought out by the agent. While desire is related to the amount of MME needed to reach satiation, it can also incorporate the seeking of additional MME for recreational use (i.e., euphoria) or increased MME related to tolerance. For an opioid-naïve user, *desire* will be equivalent to the amount of MME needed to treat acute or chronic pain.

Tolerance is assumed to be 0 for all agents upon initiation of opioid or heroin use. Once a user has been exposed to opioids, the *desire* state variable will change over time as a function of tolerance. *Desire*= (*tolerance*desire-tolerance-ratio*) where *desire-tolerance-ratio* is ranged between 1.00 and 1.50.

Patients develop *tolerance* (i.e., the dose needed to achieve satiation) over time depending on the prescribed dose, and some patients will probabilistically become non-adherent, taking a higher dose than prescribed. Tolerance initiates at 0 for all agents but builds over time depending on the amount of opioids taken and a calibrated parameter called *tolerance-inertia*. *Tolerance* is set to (*tolerance* + (1 - *tolerance-inertia*) * (*opioids-to-take* – tolerance)) where *tolerance-inertia* ranges between 0.9 and 1.0. *Opioids-to-take* is equivalent to desire, or supply (if less than desire), or prescribed dose (if compliant).

Satiation reflects the level of satiation with MME consumed during the current timestep and is bounded by 0 and 1. Agents are more likely to relapse (if within the “cessation of opioid use” use-state), more likely to acquire opioids through illicit pathways (i.e., from a dealer or friend), or take heroin/fentanyl at lower levels of satiation.

In summary, at every daily time step, agents will take MME (either prescribed PO, illicit PO, buprenorphine, or heroin) according to their desire and their internal variables, *tolerance*, *desire*, and *satiation* will then be updated accordingly. Similarly, their *opioid-supply* variable will be updated to reflect them having taken some dose of opioids. Consumption of opioids leads to a probability of overdose which increases with dose (further described in the subsection titled “Overdose”).

Table S7. Agent variables related to MME desired and consumed.

**Variable**	**Description**	**Equation or value**	**Source**
Desire	The amount of MME sought out by an individual agent	*Desire*= *required-dose-for-pain* + (*tolerance*desire-tolerance-ratio*) where *desire-tolerance-ratio* is ranged between 1.00 and 1.50	Calibrated to overdose rates observed in North Carolina from

Tolerance	The amount of MME an individual agent can consume before risk of overdose	*Tolerance = tolerance + (tolerance-inertia * (total-opioids-to-take - tolerance))*<br><br>where *tolerance-inertia* is ranged between 0.900 and 1.000 and *opioids-to-take* is equivalent to desire, supply (if less than desire), or prescribed dose (if compliant)	Calibrated
Satiation	The individual agent's level of satiation with MME consumed during that timestep, bounded by 0 and 1	For prescription compliant agents:<br><br>*Satiation= min { prescribed-dose, total-drug-supply } / desire*<br><br>Otherwise:<br><br>*Satiation = max { (total-drug-supply / desire), (satiation * 0.95) }*	Assumed

Opioid Use States

The model simulates transitions through five *use-states*: “prescription compliant”, “prescription noncompliant”, “prescription opioid use disorder”, “heroin use disorder”, and “in treatment and/or cessation of opioid use”. Each use-state is associated with a behavior within the model and changes in MME desired (**Table S8**).

Table S8. Summary of use-states in model.

Use-state	Description	Model Actions
Prescription compliant	Individuals who take PO as prescribed	- Request PO from physician or emergency department<br>- Receive PO from pharmacy or emergency department<br>- Take PO based on *desire* (set to amount needed to treat pain, function of tolerance over time)<br>- Probability of becoming non-compliant to prescribed dose
Prescription non-compliant	Individuals who take PO not as prescribed (higher MME consumed per day)	- Request PO from physician or emergency department (licit) or request PO from friends or dealer (illicit)<br>- Receive PO from pharmacy, emergency department, friend, or dealer<br>- Take PO based on *desire* (set to amount needed to treat pain, function of tolerance over time)<br>- Probability of initiating heroin use when *desire* exceeds 100 MME threshold
Prescription opioid use disorder	Individuals who take PO not as prescribed (higher MME consumed per day)	- Request PO from physician or emergency department (licit) or request PO from friends or dealer (illicit)<br>- Receive PO from pharmacy, emergency department, friend, or dealer<br>- Take PO based on *desire* (set to amount needed to treat pain, function of tolerance over time)<br>- Probability of initiating heroin use when *desire* exceeds 100 MME threshold<br>- Reduced likelihood of ceasing opioid use compared to those with non-compliance<br>- 25% annual probability of seeking treatment for OUD
Heroin use disorder	Individuals who consume heroin, can also still use PO (as prescribed or non-compliant)	- Request PO from physician or emergency department (licit) or request heroin or PO from friends or dealer (illicit)<br>- Receive PO or heroin from pharmacy, emergency department, friend, or dealer<br>- Take heroin or PO based on *desire* (set to amount needed to treat pain, function of tolerance over time)<br>- 25% annual probability of seeking treatment for OUD
In treatment and/or cessation of opioid use	Individuals with a history of opioid use who no longer actively use PO or heroin	- No longer request or use PO or heroin, overdose still possible but consumption of PO or heroin not simulated<br>- Opioid and heroin supply reset to 0<br>- Tolerance reset to 0<br>- Probability of relapse (will return to most recent use-state) dependent on treatment modality. If not receiving an MOUD, will relapse depending on the following formula:<br>$$P = r - 0.99rs$$<br>For *s*, saturation, and *r*, the maximum relapse probability parameter

If a patient’s *desire* for opioids exceeds his or her supply, the patient may transition from the “prescription compliant” to “prescription non-compliant” use state and visit additional physicians,

visit an emergency department, or buy from another patient or a dealer to take more MME than prescribed. In addition, agents may initiate heroin use if heroin is available and their *desire* reaches a MME threshold of 100 MME. The main progression of patients, based on the pathway described by Cicero et al.[24], is stochastic.

Movement between different states is determined through a transition probability matrix, informed by the literature and expert opinion.[24,25] We assume that between 8-16% of individuals prescribed an opioid will engage in misuse of an opioid (i.e., take more than the prescribed dose, seek out additional opioids) and between 2-14% of those prescribed an opioid will develop opioid dependence.[25] The probability that an agent initiates heroin use is a function of their *desire* and if heroin is available within their friend network (either a friend has heroin or is connected to a dealer). Heroin initiation is modeled as a function where an agent will begin to use heroin with a 50% annual probability when their *desire* is equivalent to 100 MME and heroin is available within their network.

Once agents are within the “opioid use disorder” or “heroin use disorder” *use-states*, agents have a 25% annual probability of seeking treatment and 60% successfully connect with treatment. Those who successfully engage in treatment, will be classified as within the “in treatment and/or cessation of opioid use” state as we do not explicitly model the uptake of PO or heroin for these agents. Agents within the “in treatment and/or cessation of opioid use” use-state will have their opioid and heroin supply variables reset to 0 and their *tolerance* variable reset to 0. Notably, we implement a probability of opioid overdose informed by the peer-reviewed literature to reflect that some agents will use opioids while engaged in treatment; however, we do not simulate the uptake of opioids while in this use-state. Treatment is further described in the subsection titled “Treatment for Opioid Use Disorder”.

Agents within the “in treatment and/or cessation of opioid use” *use-states* will probabilistically relapse depending on treatment modality (no MOUDs, buprenorphine, methadone, or naltrexone) to their most current state of active use. Agents with a low level of *satiation* (ranges from 0-1, informed by *desire* and driven by experiencing chronic or acute pain) are more likely to relapse than agents with a high level of *satiation*. In this manner, our model assumes that agents experiencing untreated chronic or acute pain are more likely to relapse.

At model initialization, we assume that a certain percentage of the population is already within selected use states (e.g., OUD, in treatment for OUD). Transitions can be forward or backward.

Agent Networks

Agents are connected via links—each agent has links to one or more physicians, one or more friends who may be other patients or dealers (*min-num-friends* (0 to 10) and *average-num-friends* (0 to 10) are set on interface), and a pharmacy. Opioids are prescribed (physicians only) and opioids or heroin/fentanyl are distributed (pharmacy, emergency departments, friends, and dealers) through these links. Below we describe the networks to distribute opioids, heroin/fentanyl, and diverted buprenorphine.

Networks of illicit opioids and heroin/fentanyl

Edges are generated between agents to reflect the friend and dealer networks through which prescription opioids and heroin/fentanyl are shared or sold. Prescription opioids (POs) are dispensed by pharmacies or emergency departments. POs can be taken as prescribed or misused. POs and heroin/fentanyl are sold by dealers or shared by friends or peers. For those misusing prescription opioids, we assume that 66% received the PO from a friend or peer, 22% from a physician, 5% from a dealer, 4% from an emergency department, and 3% from multiple physicians.[26]

Friend or peer networks are parameterized with a minimum of 0 and mean number of 2 for all agents. Networks change over time as agents can add new dealers to their social network. Adaptation takes the form of agents adding new agents who can share opioids, heroin, or diverted buprenorphine to their social network. In short, if person a_0 receives drugs from a non-dealer friend a_1, who buys from a dealer d , then a_1 will probabilistically introduce a_0 to d , thereby allowing dealers to increase their connectedness within networks. A similar process occurs for agents seeking heroin from dealers or diverted buprenorphine from dealers or friends.

Agents can "sense" the quantity of their friends' drug supply. This is analogous to some form of communication (e.g., instant messaging). This "sensing" capability is used to identify friends who might share drugs with the agent, should the agent have an inadequate supply to meet their own needs. Agents will only receive a single day's worth of opioids or heroin from a friend (vs. a dealer from whom they can receive up to seven days' worth). Heroin initiation is modeled as a function where an agent will begin to use heroin with a 50% annual probability when their desire is equivalent to 100 MME and heroin is available within their network.

Buprenorphine Diversion

If an agent is receiving buprenorphine as an MOUD, we simulate the creation of buprenorphine sharing networks (**Table S9**). Buprenorphine diversion is defined as the unauthorized rerouting of prescription buprenorphine to someone other than for whom it was intended either voluntarily or involuntarily and with or without exchange of money or goods.[27]

Within the ABM, we simulate diversion through the following steps: 1) an agent enters into buprenorphine treatment within the "cessation of opioid use" *use-state*, 2) the agent diverts a certain number of doses from their prescribed supply (typically 1-2 doses per week) to individuals with opioid misuse and/or OUD within their peer network, 3) the agent receiving the diverted buprenorphine will replace their typical opioid use (i.e., use of heroin/fentanyl, and/or non-medical use of prescription opioids) with the buprenorphine on the day they receive the buprenorphine. Agents seeking diverted buprenorphine will receive 1 day dose from friends or peers and up to 3 days from a dealer. We do not simulate the exchange of money, good, or services for diverted buprenorphine or the sale of illicitly manufactured buprenorphine. We

assume that the agent prescribed buprenorphine and diverting their supply does not face any negative consequences related to diversion (i.e., increase in desire, punitive measures from treatment facility, etc). We assume that on the day the diverted buprenorphine is consumed, agents have the same risk of overdose as agents taking prescribed buprenorphine.

We informed the probability of sharing or selling buprenorphine, conditional on having a current prescription, based on reports from Lofwall et al. and Kenney et al.[1,28] We assume that 10% of agents who are non-compliant with opioids or use heroin/fentanyl will also use diverted buprenorphine based on the literature.[29-32] For the 10% of agents with illicit PO or heroin use who also use diverted buprenorphine, we assume that the agent has a 15% daily probability of using diverted buprenorphine vs. heroin/fentanyl or PO based on Carlson et al.[33] We assume that 80% of diverted buprenorphine is sourced from a friend rather than a dealer.[28,34] We incorporate assortative mixing within the model so that agents who have ever had a buprenorphine prescription are more likely to develop connections with other agents who have ever had a buprenorphine prescription or have used diverted buprenorphine. Due to the persistent stigma associated with the use of MOUDs, we implemented this assortative mixing parameter to simulate the growth of networks of individuals who hold less stigma towards the use of buprenorphine. Additionally, this can reflect friends introducing their network to friends who are prescribed and willing to share or sell buprenorphine.

Table S9. Parameters related to buprenorphine diversion within base case scenario.

Parameter	Value	Source
Probability of sharing/selling buprenorphine conditional on being prescribed	50%	Lofwall 2015[1], Kenney 2017[28]
Probability of using diverted buprenorphine conditional on using illicit opioids	10%	Genberg 2013[29], Yokell 2011[30], Fox 2015[31], Novak 2015[32]
Probability of using diverted buprenorphine vs. illicit opioid conditional on having access to diverted buprenorphine	15%	Carlson et al.[33]
Probability of sourcing diverted buprenorphine from a friend/peer vs. dealer	80%	Kenney 2017[28], Larance et al.[34]
Probability of overdose while using diverted buprenorphine	2.08 per 100 person-years	Morgan et al.[35]
Assortative mixing parameter for agents who have been prescribed buprenorphine and/or have used diverted buprenorphine	45%	Assumed

Dealers

The "dealer" location type includes entities that can sell illicit PO, heroin, or diverted buprenorphine. State variables associated with dealers primarily track their supply of available drugs (**Table S10**). Due to lack of data on the amount of MME within an individual dealer's supply at any one time, we informed our estimates based on expert opinion and assumptions. Similarly, limited information exists on the exact percentage of heroin available adulterated with fentanyl. However, the percentage is likely high and growing. In addition, fentanyl-laced opioids in pill form are increasingly available through illicit markets. Based on data reported in Ohio using DEA drug seizure data and self-reported discernment of fentanyl (as confirmed by

fentanyl test strips) in North Carolina as well as conversations with Dr. Jon Zibbell, we assume all heroin is currently contaminated with some amount of fentanyl.[36,37]

When an agent visits a dealer, they make a request for 7 days-worth of their desired MME. If the dealer has not provided the agent's desired quantity, they seek a new dealer from within their friends' network.

Table S10. State variables for dealers

**Variable**	**Description**	**Value, distribution**	**Source**
Mean-opioid-supply	Mean amount of prescription opioids in MME that the dealer has at any one point	400, exponential	Expert opinion
Mean-heroin-supply	Mean amount of heroin in MME that a dealer has at any one point	4000, exponential	Expert opinion

Treatment for Opioid Use Disorder

Parameters related to cessation of opioid use and treatment for opioid use disorder are summarized in **Table S11**. To inform the likelihood that an agent seeks treatment for opioid use disorder, we used survey data. In 2019 NSDUH public-use data, 8.08% of individuals who reported misusing opioids in the past year reported either receiving drug treatment or that they needed/made effort to get treatment/additional treatment in the past year. This is likely an undercount since it excludes individuals who may have sought treatment due to court mandate, family or job pressure, or other reasons. Therefore, we sought out additional studies to inform this probability. In a survey of 137 individuals accessing syringe service program services, Fox et al. report that more than half (57%) were interested in buprenorphine treatment.[38] National Epidemiologic Survey on Alcohol and Related Conditions (NESARC) data from 2004-2005 reported by Blanco et al. that for individuals with a prescription opioid use disorder (n=623) there was a 11.1% probability during the first year of disorder onset, 24.5% during the first ten years, and 42.4% ever of seeking treatment.[39] The median delay to treatment-seeking was nearly 4 years. Due to the wide ranges reported, we assumed that the annual probability of seeking treatment for individuals with prescription opioid misuse or heroin use was 25% and ranged from 5-45%.

Considering significant barriers related to treatment access, only a proportion of those agents seeking treatment enter treatment. We informed the number of agents entering and currently within treatment for opioid use disorder with data from The National Survey of Substance Abuse Treatment Services (N-SSATS). Due to significant disruptions to substance use treatment facilities due to the COVID-19 pandemic, we utilized the 2019 N-SSATS survey for North Carolina. In 2019, there were 214 facilities providing methadone/buprenorphine maintenance or naltrexone treatment on March 29, 2019. N-SSATS reports statistics for both OTP (federally-certified Opioid Treatment Program) and non-OTP facilities that provide MAT. In total, 24,227 patients received MAT in North Carolina in 2019 (n=17,139 within OTP facilities and 7,088 in non-OTP facilities). We also used the N-SSATS to calculate the probability of entering different types of treatment (methadone vs. buprenorphine vs. naltrexone).

We based the number of individuals in North Carolina entering treatment on a news article from 2019 reporting that in North Carolina "the state health department collected demographic data on 10,333 people who entered substance abuse treatment over the past two years through the 21st Century Cures Act State Targeted Response to the Opioid Crisis Grants." We divided this by half to equal 5,167 North Carolina residents entering treatment per year.

Upon entering the “in treatment and/or cessation of opioid use” *use-state*, an agent will discard all opioids in their possession. Their tolerance immediately declines to 0 and they are probabilistically assigned to different types of treatment. Informed by the literature[40-42], a probability of relapse is set depending on treatment modality (no MOUD, methadone, buprenorphine, naltrexone) and they probabilistically relapse.[43-45] Upon relapse, they return to their most recent active use state.

Table S11. Parameters related to treatment for opioid use disorder

Parameter	Description	Value, distribution	Source
treatment-rate	Annual probability of seeking treatment for agents who misuse opioids or use heroin	25% (5-45%)	NSDUH 2019, Fox et al.[38], Blanco et al.[39]

Number of individuals starting treatment per year	Estimated number of individuals entering treatment for opioid use disorder per year	5167	Knopf[46]
PO disorder treatment proportion	Number of people with an opioid use disorder that are currently in treatment	24227	N-SSATS 2019
methadone-prob	Probability of receiving methadone once somebody starts treatment	0.59	N-SSATS 2019
buprenorphine-prob	Probability of receiving buprenorphine once somebody starts treatment	0.38	N-SSATS 2019
naltrexone-prob	Probability of receiving naltrexone once somebody starts treatment	0.03	N-SSATS 2019
methadone-OD-prob	Overdose rate for an agent actively taking methadone	2 per 100 person-years	Sordo et al.[47]
buprenorphine-OD-prob	Overdose rate for an agent actively taking buprenorphine	2.08 per 100 person-years	Morgan et al.[35]
naltrexone-OD-prob	Overdose rate for an agent with a recent (less than one month old) injectable naltrexone	3.85 per 100 person-years	Morgan et al.[35]
methadone-max-relapse-prob	Annual probability of treatment cessation, agent goes back to most recent active use state	0.55 by one year	Timko et al.[44], Soyka et al.[43]
buprenorphine-max-relapse-prob	Annual probability of treatment cessation, agent goes back to most recent active use state	0.31 by one month, 0.735 by one year	Morgan et al.[45]
naltrexone-max-relapse-prob	Annual probability of treatment cessation, agent goes back to most recent active use state	0.52 by one month, 0.95 by one year	Morgan et al.[45]

Overdose

Agents can non-fatally or fatally overdose when consuming PO or heroin/fentanyl. The probability of overdose varies dependent on the dose (in MME), and whether the consumed opioid is heroin. The probability of dying conditional on experiencing overdose varies depending on if naloxone is available and if heroin/fentanyl was consumed. Parameters and sources are summarized in **Table S12**.

Table S12. Parameters related to non-fatal and fatal overdose.

Parameter	Description	Value, distribution	Source
Baseline probability of overdose	Algorithm of probability of overdose by MME dose	Varies	Dunn et al.[48], Calibrated to NCDHHS
Heroin-OD-rate-multiplier	Multiplier of risk of overdose related to use of heroin compared to someone solely using prescription opioids (calibrated to reflect NC OD data)	1.7	Calibrated to NCDHHS data
Death-probability	Probability of fatal overdose, conditional on experiencing overdose	0.17	Calibrated to NCDHHS data
Heroin-OD-death-multiplier	Multiplier of risk of fatal overdose related to use of heroin compared to someone solely using prescription opioids (calibrated to reflect NC OD data)	1.5	Calibrated to NCDHHS data
Naloxone-availability	Probability of naloxone being available to any agent	27.6%	https://naloxonesaves.org/community-distribution-of-naloxone/, NCDHHS
Naloxone-effectiveness	Probability of reversal after use of naloxone during an overdose event	87.5% (75-100%)	Clark et al.[49]

The amount of MME consumed by an agent is informed by their *desire* internal state variable as described earlier. The probability of overdose is calibrated to approximate NCDHHS data and is informed by overdose data reported by Dunn et al.[48] This algorithm gives the probability of an overdose given dosage amount. If an agent is not on treatment, their probability of overdosing is given by a linear model fit to overdose data (converted to annual per person rates). Otherwise, we use fixed parameters to estimate their probability of overdose as reported within the subsection “Treatment for Opioid Use Disorder”.

Use of heroin increases the probability of overdose by 70% compared to users of PO only as we assume that agents using heroin will imprecisely take their desired amount and heroin varies in strength across batches. We assume that all heroin is contaminated with fentanyl. Agents using heroin first draw from any heroin supply on hand before drawing from their opioid supply.

The baseline probability of fatal overdose conditional on overdose is calibrated to NCDHHS data, in conjunction with other model parameters (e.g., naloxone availability), and is increased for agents using heroin and decreased for agents with access to naloxone.

Using NC DHHS data, we assume that agents using heroin have 50% increased risk of death. We informed the likelihood that agents have access to naloxone based on the number of community-distributed intranasal naloxone kits given out in North Carolina and the number of naloxone reversals reported in one year. A non-profit organization (https://naloxonesaves.org/community-distribution-of-naloxone/) reported that 113,000 kits were

distributed in North Carolina from 2016-2019. The NC DHHS reported in a presentation to the public that from 2019-2020, there were 8,666 cases of naloxone reversals over one year reported by the NC Department of Health and Human Services. This was an increase with the previous years (4,817 from 2018-2019 and 2,660 in 2017-2018). We calculated the probability that a naloxone kit is available to a user upon overdose as the number of kits available (113,000) by the average number of active users at any one point in time during a ten-year model run (n=408,000) to estimate that a user in North Carolina has a 27.6% probability of having naloxone available upon overdose. If naloxone is used, we assume that 87.5% of users will successfully reverse the overdose.[49]

Model Calibration

To calibrate the model, we evaluated the ABM by its ability to reproduce patterns related to non-fatal and fatal overdose, treatment engagement, and additional purpose-specific patterns. In total, 75 parameters were used in calibration with a mean-squared error (MSE) approach using ten years of reported opioid-related overdose deaths in North Carolina from 2010 to 2019 (**Figure S3**). To do so, we used the simulated annealing algorithm implemented in BehaviorSearch.

Figure S3. Annual number of opioid overdose deaths observed in North Carolina (blue line) and simulated by the ABM (black line).

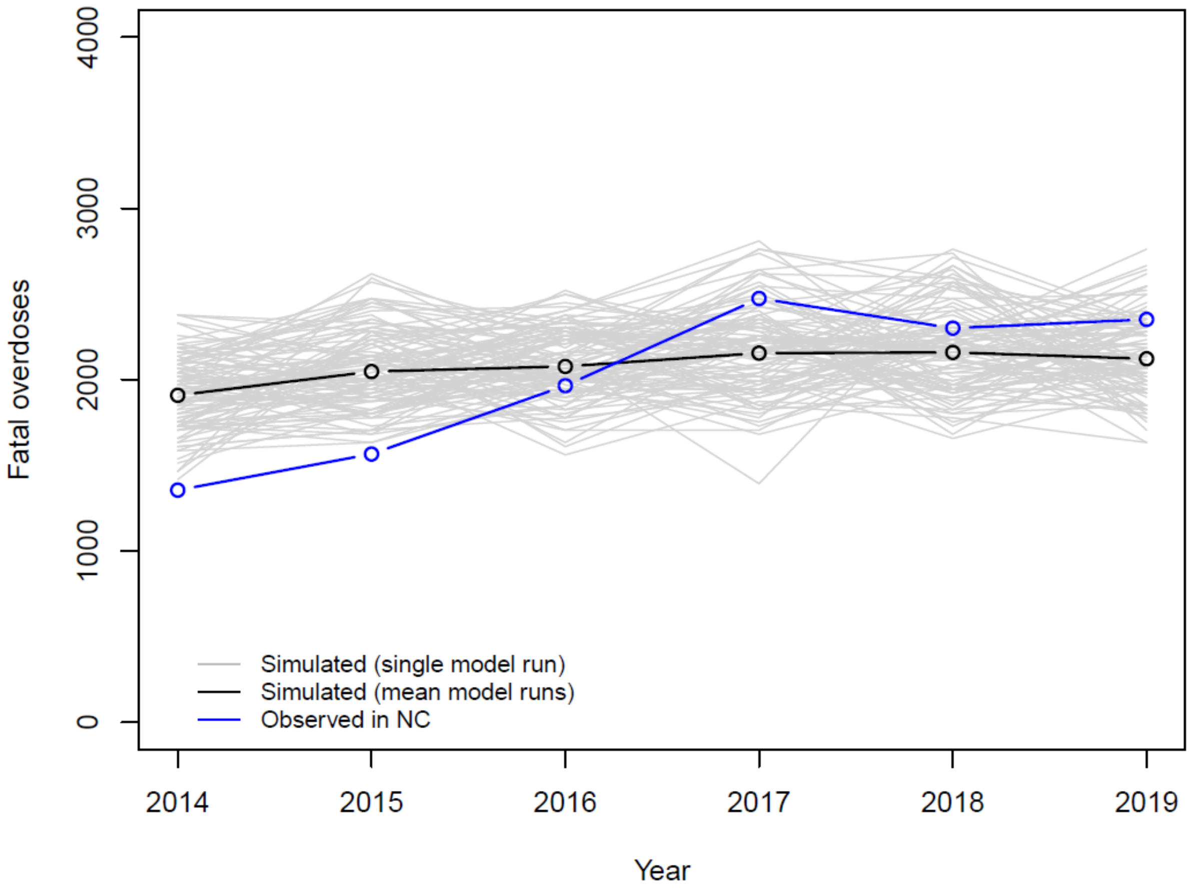

References

1. Lofwall MR, Walsh SL. A review of buprenorphine diversion and misuse: the current evidence base and experiences from around the world. (1935-3227 (Electronic)).
2. Mikosz CA, Zhang K, Haegerich T, et al. Indication-Specific Opioid Prescribing for US Patients With Medicaid or Private Insurance, 2017. *JAMA Network Open.* 2020;3(5):e204514-e204514.
3. Howard R, Fry B, Gunaseelan V, et al. Association of Opioid Prescribing With Opioid Consumption After Surgery in Michigan. *JAMA Surgery.* 2019;154(1):e184234-e184234.
4. Dowell D, Haegerich TM, Chou R. CDC Guideline for Prescribing Opioids for Chronic Pain—United States, 2016. *JAMA.* 2016;315(15):1624-1645.
5. Mundkur ML, Franklin JM, Abdia Y, et al. Days' Supply of Initial Opioid Analgesic Prescriptions and Additional Fills for Acute Pain Conditions Treated in the Primary Care Setting - United States, 2014. *MMWR Morb Mortal Wkly Rep.* 2019;68(6):140-143.
6. Chronic pain and high-impact chronic pain among U.S. adults, 2019, (2020).
7. Mathieson S, Wertheimer G, Maher CG, et al. What proportion of patients with chronic noncancer pain are prescribed an opioid medicine? Systematic review and meta-regression of observational studies. *Journal of Internal Medicine.* 2020;287(5):458-474.
8. Naliboff BD, Wu SM, Schieffer B, et al. A Randomized Trial of 2 Prescription Strategies for Opioid Treatment of Chronic Nonmalignant Pain. *The Journal of Pain.* 2011;12(2):288-296.
9. Health UDo, Services H. HHS guide for clinicians on the appropriate dosage reduction or discontinuation of long-term opioid analgesics. *Washington, DC: US Department of Health and Human Services.* 2019.
10. Food, Administration D. FDA identifies harm reported from sudden discontinuation of opioid pain medicines and requires label changes to guide prescribers on gradual, individualized tapering. *Food and drug administration website Available at https://www fda gov/drugs/drug-safety-and-availability/fda-identifies-harm-reported-sudden-discontinuationopioid-pain-medicines-and-requires-label-changes Updated April.* 2019;9.
11. Barnett ML, Olenski AR, Jena AB. Opioid-Prescribing Patterns of Emergency Physicians and Risk of Long-Term Use. *New England Journal of Medicine.* 2017;376(7):663-673.
12. Borrelli EP, Morphis B, Youssef R, Laura C, Hallowell BD, Bratberg J. Concurrent Utilization of Prescription Opioids and Non-opioid Controlled Substances: Rhode Island Prescription Drug Monitoring Program, 2018. *RI Med J.* 2013;103:53-58.
13. Hung A, Bush C, Greiner M, et al. Risk Factors and Outcomes of Opioid Users with and Without Concurrent Benzodiazepine Use in the North Carolina Medicaid Population. *Journal of Managed Care & Specialty Pharmacy.* 2020;26(2):169-175.
14. Dayer LE, Breckling MN, Kling BS, Lakkad M, McDade ER, Painter JT. Association of the “CDC Guideline for Prescribing Opioids for Chronic Pain” With

Emergency Department Opioid Prescribing. *The Journal of Emergency Medicine.* 2019;57(5):597-602.
15. Sun BC, Charlesworth CJ, Lupulescu-Mann N, et al. Effect of Automated Prescription Drug Monitoring Program Queries on Emergency Department Opioid Prescribing. *Annals of Emergency Medicine.* 2018;71(3):337-347.e336.
16. Control CfD, Prevention. Annual Surveillance Report of Drug-Related Risks and Outcomes—United States Surveillance Special Report. Centers for Disease Control and Prevention, US Department of Health and Human Services. Published November 1, 2019. In: Accessed 2/1/21 at https://www. cdc. gov/drugoverdose/pdf/pubs/2019-cdc-drug …; 2019.
17. Gallagher R. Multiple opioids in pain management. *Canadian Family Physician.* 2007;53(12):2119-2120.
18. Goldstein A. *Addiction: From biology to drug policy.* Oxford University Press; 2001.
19. Hayden JA, Ellis J, Asbridge M, et al. Prolonged opioid use among opioid-naive individuals after prescription for nonspecific low back pain in the emergency department. *Pain.* 2021;162(3):740-748.
20. Hoffer LD. *Junkie business: The evolution and operation of a heroin dealing network.* University of Colorado at Denver; 2002.
21. Koob GF, Volkow ND. Neurocircuitry of addiction. *Neuropsychopharmacology.* 2010;35(1):217-238.
22. Preiss A, Berghammcr A, Bobashev G. Virtual Opioid User: Reproducing Opioid Use Phenomena with a Control Theory Model. Paper presented at: 2022 Winter Simulation Conference (WSC)2022.
23. Koob G, Le Moal M. Neurobiological theories of addiction. *Neurobiology of addiction.* 2006:378-428.
24. Cicero TJ, Ellis MS, Surratt HL, Kurtz SP. The Changing Face of Heroin Use in the United States: A Retrospective Analysis of the Past 50 Years. *JAMA Psychiatry.* 2014;71(7):821-826.
25. The Effectiveness and Risks of Long-Term Opioid Therapy for Chronic Pain: A Systematic Review for a National Institutes of Health Pathways to Prevention Workshop. *Annals of Internal Medicine.* 2015;162(4):276-286.
26. Lipari RN, Hughes A. How people obtain the prescription pain relievers they misuse. *The CBHSQ report.* 2017.
27. LARANCE B, DEGENHARDT L, LINTZERIS N, WINSTOCK A, MATTICK R. Definitions related to the use of pharmaceutical opioids: Extramedical use, diversion, non-adherence and aberrant medication-related behaviours. *Drug and Alcohol Review.* 2011;30(3):236-245.
28. Kenney SR, Anderson BJ, Bailey GL, Stein MD. The relationship between diversion-related attitudes and sharing and selling buprenorphine. *Journal of Substance Abuse Treatment.* 2017;78:43-47.
29. Genberg BL, Gillespie M, Schuster CR, et al. Prevalence and correlates of street-obtained buprenorphine use among current and former injectors in Baltimore, Maryland. *Addictive Behaviors.* 2013;38(12):2868-2873.

30. A. Yokell M, D. Zaller N, C. Green T, D. Rich J. Buprenorphine and Buprenorphine/Naloxone Diversion, Misuse, and Illicit Use: An International Review. *Current Drug Abuse Reviews.* 2011;4(1):28-41.
31. Fox AD, Chamberlain A, Frost T, Cunningham CO. Harm Reduction Agencies as a Potential Site for Buprenorphine Treatment. *Substance Abuse.* 2015;36(2):155-160.
32. Novak SP, Wenger L, Lorvick J, Kral A. The misuse, abuse and diversion of opioid replacement therapies among street abusers. *Drug and Alcohol Dependence.* 2015;146:e54.
33. Carlson RG, Daniulaityte R, Silverstein SM, Nahhas RW, Martins SS. Unintentional drug overdose: Is more frequent use of non-prescribed buprenorphine associated with lower risk of overdose? *International Journal of Drug Policy.* 2020;79:102722.
34. Larance B, Degenhardt L, Lintzeris N, et al. Post-marketing surveillance of buprenorphine-naloxone in Australia: Diversion, injection and adherence with supervised dosing. *Drug and Alcohol Dependence.* 2011;118(2):265-273.
35. Morgan JR, Schackman BR, Weinstein ZM, Walley AY, Linas BP. Overdose following initiation of naltrexone and buprenorphine medication treatment for opioid use disorder in a United States commercially insured cohort. *Drug and Alcohol Dependence.* 2019;200:34-39.
36. Zibbell JE, Peiper NC, Duhart Clarke SE, et al. Consumer discernment of fentanyl in illicit opioids confirmed by fentanyl test strips: Lessons from a syringe services program in North Carolina. *International Journal of Drug Policy.* 2021;93:103128.
37. Zibbell JE, Aldridge AP, Cauchon D, DeFiore-Hyrmer J, Conway KP. Association of Law Enforcement Seizures of Heroin, Fentanyl, and Carfentanil With Opioid Overdose Deaths in Ohio, 2014-2017. *JAMA Network Open.* 2019;2(11):e1914666-e1914666.
38. Fox AD, Shah PA, Sohler NL, Lopez CM, Starrels JL, Cunningham CO. I Heard About It from a Friend: Assessing Interest in Buprenorphine Treatment. *Substance Abuse.* 2014;35(1):74-79.
39. Blanco C, Iza M, Schwartz RP, Rafful C, Wang S, Olfson M. Probability and predictors of treatment-seeking for prescription opioid use disorders: A National Study. *Drug and Alcohol Dependence.* 2013;131(1):143-148.
40. Woody GE, Poole SA, Subramaniam G, et al. Extended vs Short-term Buprenorphine-Naloxone for Treatment of Opioid-Addicted Youth: A Randomized Trial. *JAMA.* 2008;300(17).
41. Mattick RP, Breen C, Kimber J, Davoli M. Methadone maintenance therapy versus no opioid replacement therapy for opioid dependence. *Cochrane Database of Systematic Reviews.* 2009(3).
42. Tanum L, Solli KK, Latif Z-e-H, et al. Effectiveness of Injectable Extended-Release Naltrexone vs Daily Buprenorphine-Naloxone for Opioid Dependence: A Randomized Clinical Noninferiority Trial. *JAMA Psychiatry.* 2017;74(12):1197-1205.
43. Soyka M, Zingg C, Koller G, Kuefner H. Retention rate and substance use in methadone and buprenorphine maintenance therapy and predictors of outcome:

results from a randomized study. *The The International Journal of Neuropsychopharmacology.* 2008;11(5):641-653.
44. Timko C, Schultz NR, Cucciare MA, Vittorio L, Garrison-Diehn C. Retention in medication-assisted treatment for opiate dependence: A systematic review. *Journal of Addictive Diseases.* 2016;35(1):22-35.
45. Morgan JR, Schackman BR, Leff JA, Linas BP, Walley AY. Injectable naltrexone, oral naltrexone, and buprenorphine utilization and discontinuation among individuals treated for opioid use disorder in a United States commercially insured population. *Journal of Substance Abuse Treatment.* 2018;85:90-96.
46. Knopf T. N.C. uses new federal money to get people into drug treatment, but most of them are white *North Carolina Health News*2019.
47. Sordo L, Barrio G, Bravo MJ, et al. Mortality risk during and after opioid substitution treatment: systematic review and meta-analysis of cohort studies. *BMJ.* 2017;357:j1550.
48. Dunn KM, Saunders Kw Fau - Rutter CM, Rutter Cm Fau - Banta-Green CJ, et al. Opioid prescriptions for chronic pain and overdose: a cohort study. (1539-3704 (Electronic)).
49. Clark AK, Wilder CM, Winstanley EL. A Systematic Review of Community Opioid Overdose Prevention and Naloxone Distribution Programs. *Journal of Addiction Medicine.* 2014;8(3).
